# Identification of coagulation-related biomarkers in osteoarthritis and immune infiltration analysis based on bioinformatics

**DOI:** 10.1186/s41065-025-00585-3

**Published:** 2025-10-27

**Authors:** Linyuwei He, Zhihong Ou, Boyuan Qiu, Siwen Tong, Chu Liu, Pengwei Zhou, Zhixue Ou

**Affiliations:** 1 The Graduate School, Guangxi University of Traditional Chinese Medicine, Nanning, Guangxi 530200 China; 2 Clinical Laboratory, Guilin Municipal Hospital of Traditional Chinese Medicine, Guilin, Guangxi 541000 China; 3 Joints and Sports Medicine Department, Guilin Municipal Hospital of Traditional Chinese Medicine, No. 2, Lingui Road, Xiangshan District, Guilin, Guangxi 541000 China

**Keywords:** OA, Coagulation-Related genes (CRGs), Machine learning (ML), Immune infiltration, Biomarkers

## Abstract

**Background:**

Osteoarthritis (OA) is a common degenerative disorder characterized primarily by articular cartilage degradation and chronic inflammation. Although direct evidence elucidating the specific mechanisms underlying the coagulation-immune axis in OA remains limited, emerging studies have suggested a potential link.

**Methods:**

Four microarray datasets were retrieved from the Gene Expression Omnibus (GEO) database. Then, differentially expressed genes (DEGs) (|log₂FC| ≥ 1, *P* < 0.05) were identified. Gene Set Enrichment Analysis (GSEA), Gene Ontology (GO), and Kyoto Encyclopedia of Genes and Genomes (KEGG) enrichment analyses were performed on these DEGs. Molecular Signatures Database (MsigDB) coagulation genes were intersected with DEGs to identify coagulation-related DEGs. Then, hub genes were determined using multiple Machine learning (ML) algorithms, Least Absolute Shrinkage and Selection Operator (LASSO), Support Vector Machine-Recursive Feature Elimination (SVM-RFE), and Random Forest (RF). Diagnostic performance of these genes was evaluated via a nomogram and ROC analysis (AUC). Immune cell infiltration was assessed with CIBERSORT. The expression of hub genes was validated in vitro via real-time qPCR and Western blot (WB).

**Results:**

Based on 103 samples across four datasets, 294 DEGs were identified. Gene set enrichment analyses (GSEA, GO, KEGG) revealed significant enrichment of these genes in immune- and coagulation-related pathways in OA. Intersecting MsigDB coagulation genes with DEGs yielded nine coagulation-associated DEGs. Based on four distinct ML algorithms, six hub genes were selected: Fibroblast activation protein (FAP), Cathepsin H (CTSH), matrix metalloproteinase 1 (MMP1), matrix metalloproteinase 9 (MMP9), Complement component 6 (C6), MAF Basic Leucine Zipper Transcription Factor F (MAFF). These hub genes demonstrated high diagnostic accuracy according to ROC analysis. Immune infiltration analysis showed significant differences between OA and normal samples. M0 macrophages, plasma cells, and γδ T cells were elevated in OA, while activated mast cells and resting memory CD4⁺ T cells were decreased. The qPCR and WB results corroborated the ML findings: in the interleukin-1β (IL-1β)-treated group, FAP, MMP1, MMP9, and CTSH were significantly upregulated, while MAFF and C6 were markedly downregulated.

**Conclusions:**

This study, based on publicly available GEO datasets, identified six potential diagnostic biomarkers for OA: FAP, CTSH, MMP1, MMP9, C6, and MAFF. These findings highlight the potential involvement of coagulation-immune interactions in OA pathogenesis and offer novel insights into the molecular mechanisms and diagnostic strategies for the disease.

## Introduction

Osteoarthritis (OA) is a complex joint disorder with a pathogenesis that remains incompletely elucidated. Recent studies have demonstrated that inflammatory and immune responses play central roles in the pathophysiological processes of OA. Moreover, intimate crosstalk exists between the coagulation system and immune responses, providing a theoretical framework for investigating the association between OA and coagulation pathways [1, 2].

Biomarkers hold significant value in OA research. Cartilage oligomeric matrix protein (COMP), a well-established marker of cartilage degradation, plays a pivotal role in maintaining the structural integrity of cartilage [3, 4]. Chemokine CCL2 has also been proposed as a potential biomarker of OA, as its levels are markedly elevated in patients with joint injury, where it promotes MMP-13 expression and exacerbates extracellular matrix degradation [5, 6]. However, these biomarkers remain limited by insufficient tissue specificity, underscoring the urgent need to identify more reliable novel biomarkers [3, 6].

The coagulation system contributes to the regulation of inflammation through multiple mechanisms. For instance, thrombin can activate protease-activated receptors (PARs), thereby modulating the release of inflammatory mediators, while factor XIIa and kallikrein in the contact activation pathway not only promote coagulation but also amplify inflammatory signaling [7, 8]. In addition, coagulation factors such as fibrinogen and D-dimer have been established as markers of a hypercoagulable state in rheumatoid arthritis (RA), suggesting a potential link between coagulation abnormalities and chronic joint diseases [9, 10]. In OA, although direct evidence remains limited, platelet activation and the release of procoagulant mediators have been implicated in aggravating synovial inflammation and cartilage degradation [1, 11]. For example, thrombin can cleave and activate IL-1α, thereby promoting thrombopoiesis and local inflammatory responses, effectively bridging coagulation processes with immune regulatory mechanisms relevant to OA [12, 13].

Our study presents a novel approach by leveraging bioinformatics tools to elucidate the interplay between immune responses and coagulation in OA. This study applies four distinct machine learning (ML) algorithms to screen differentially expressed genes (DEGs). By elucidating the mechanisms of coagulation and immunity in OA, our findings could help uncover diagnostic biomarkers and inspire new therapeutic approaches.

## Materials and methods

### Microarray data sources

The datasets GSE55235 [14], GSE77298 [15], and GSE82107 [16] were obtained from the Gene Expression Omnibus (GEO). Specifically, the first dataset [14] comprises 20 OA and 10 control samples; GSE55457 includes 23 OA and 10 control samples; GSE77298 consists of 16 OA samples and 10 control samples; and GSE82107 contains 16 OA and 10 control samples. The GSE55235 and GSE55457 datasets were generated using the GPL96 platform, while GSE77298 and GSE82107 were based on the GPL570 platform. All datasets were derived from Homo sapiens samples (Table 1). This study utilized publicly available datasets.

**Table 1:** Summary of microarray data sets from GEO

Data number	Platform information	OA group	Control group	Species
GSE55235	GPL96	20	10	Homo sapien
GSE55457		23	10	Homo sapien
GSE77298	GPL570	16	7	Homo sapien
GSE82107		10	7	Homo sapien

To eliminate batch effects and integrate the GEO datasets, batch correction was performed using the sva package in R (https://bioconductor.org/packages/release/bioc/html/sva.html). This preprocessing yielded a merged dataset comprising 69 OA samples and 34 control samples.

Using “coagulation” as the keyword, relevant gene sets were retrieved from the Molecular Signatures Database (MsigDB), and subsequently, the CRG sets identified in Gene Ontology (Biological Process), KEGG, and Hallmark collections were integrated using R to generate the CRG set. The CRG set was retrieved from the MsigDB and consisted of 221 genes (https://www.gsea-msigdb.org/gsea/msigdb/index.jsp).

### Data integration

Batch effect removal was executed via the sva, factoextra, and tinyarray R packages (https://cran.r-project.org/web/packages/tinyarray/refman/tinyarray.html). The ComBat approach was leveraged to eliminate batch effects across four integrated GEO datasets. Principal component analysis (PCA) and boxplots were employed for visualizing the effectiveness of batch effect correction.

### Differential expression analysis (DEA)

DEG analysis was conducted via the limma R package 3.60.6 [17, 18] to identify genes exhibiting differential expression between disease and control samples within the integrated dataset, thereby assessing the effects of immune-related gene expression on OA. DEGs were identified using the following criteria: an absolute log₂ fold change (|log₂FC|) >1 and an adjusted p-value (adj. P) < 0.05. Genes with log₂FC >1 and adj. *P* < 0.05 were classified as upregulated, while those with log₂FC < −1 and adj. *P* < 0.05 was considered downregulated. DEG expression patterns were visualized using volcano plots and heatmaps.

### Functional enrichment analysis

The msigdbr package (https://cran.r-project.org/web/packages/msigdbr/) in R was employed to perform Gene Set Enrichment Analysis (GSEA). The clusterProfiler R package [19] was utilized for Gene Ontology (GO) annotation [20] and Kyoto Encyclopedia of Genes and Genomes (KEGG) pathway enrichment analysis [21]. *p* < 0.05 and q < 0.05 denote statistical significance to determine statistically significant enrichment.

### Correlation analysis between CRGs and DEGs

The overlap between DEGs and a predefined set of CRGs was identified and visualized using Venn diagrams. Correlation analyses were performed using the linkET (https://github.com/Hy4m/linkET) and qcorrplot R packages. Pearson correlation coefficients were calculated with the qcorr function, while Spearman’s rank correlation was employed to assess gene-gene relationships. Correlation heatmaps and other visualizations were generated via the linkET package.

### Protein-protein interaction (PPI) network analysis

A PPI network was constructed for the intersection genes between DEGs and CRGs using the STRING database (https://cn.string-db.org/) [22].

### ML analysis

A Least Absolute Shrinkage and Selection Operator (LASSO) regression model [23] was constructed via the glmnet R package (https://CRAN.R-project.org/package=glmnet) [23], with the α parameter set to 1 [24, 25]. A five-fold cross-validation approach was employed to identify OA-related feature genes based on the optimal λ value. The e1071 and caret R packages facilitated feature selection by iteratively eliminating the least contributory variables, refining the set of OA-related characteristic genes. A random forest (RF) model was constructed via the randomForest R package. The number of decision trees was set to 1,000 [26, 27], and feature importance scores were analyzed to identify disease-associated genes. In addition, univariate logistic regression (LR) analysis was conducted for each gene using a custom function. Genes with p-values < 0.05 were retained. The corresponding log odds ratios (log[OR]) and 95% confidence intervals (CIs) were visualized via a forest plot. The Venn package was used to determine the intersection of candidate genes identified by the four ML algorithms, and a Venn diagram was generated to illustrate the overlap.

### Nomogram model construction

An LR model was constructed through the rms R package(https://cran.r-project.org/web/packages/rms/index.html), and a nomogram was derived to illustrate the contribution of each variable to disease diagnosis. The pROC R package (https://cran.r-project.org/web/packages/pROC/) was employed for creating receiver operating characteristic (ROC) curves and computing the area under the curve (AUC) to assess the predictive accuracy of the nomogram. The clinical utility of the model across threshold probabilities was evaluated through decision curve analysis (DCA) via the calibrate function.

### ROC analysis

ROC curve analysis was enabled by the pROC R package for the evaluation of the diagnostic efficacy of each key gene identified through the four ML approaches. The AUC values for each gene in the OA and control groups were computed, and individual gene ROC curves were plotted. The box plots illustrating the expression of each key gene were created utilizing the ggplot2 package (https://ggplot2.tidyverse.org) in R, with statistical significance assessed via the Wilcoxon test [28–30].

### Immuno-infiltration analysis

The CIBERSORT algorithm, a computational method based on linear support vector regression (SVR), was employed to deconvolute the relative proportions of distinct cell types from bulk gene expression profiles [31]. The CIBERSORT algorithm, in combination with the LM22 signature matrix, was employed to estimate the relative abundance of 22 immune cell types in each sample. Cell types with zero abundance across all samples were excluded to ensure valid estimates of immune cell composition.

Bar plots and box plots were generated to visualize the differences in immune cell proportions between groups. Statistical significance was determined through the Kruskal-Wallis test [32].

### qRT-PCR validation of key genes

To simulate the effects of the OA microenvironment on chondrocytes, human normal chondrocytes (C28/I2) were obtained from Wuhan Cloud-Clone Corp. and cultured in Dulbecco’s Modification of Eagle’s Medium/Ham’s F-12 Medium (DMEM/F12) medium (Wuhan SUNNCELL Biotechnology Co., Ltd.) supplemented with 10% fetal bovine serum (FBS) and 1% penicillin-streptomycin at 37 °C in a humidified atmosphere containing 5% CO₂. Subsequently, C28/I2 cells were divided into two groups: the control group (NC) and the experimental group (OA). To mimic OA conditions, OA group cells were treated with 10 ng/mL IL-1β for 24 h [33], while NC group cells were maintained in standard culture medium. After 48 h of culture, RNA was extracted from both groups, followed by cDNA synthesis. Total RNA was extracted using TRIzol reagent, quantified with a NanoDrop spectrophotometer, and 500 ng of total RNA per reaction was used. Reverse transcription was performed in strict accordance with the HiScript III RT SuperMix for qPCR (+ gDNA wiper) protocol (Vazyme, #R323-01): 5 µL of RNA template (500 ng total RNA, brought to 12 µL with RNase-free water) was mixed with 4 µL of 4× gDNA Wiper Mix, incubated at 42 °C for 2 min to remove genomic DNA, followed by the addition of 4 µL of 5× HiScript III qRT SuperMix to bring the total reaction volume to 20 µL. The reaction was incubated at 37 °C for 15 min for cDNA synthesis, then heated at 85 °C for 5 s to inactivate reverse transcriptase. The resulting cDNA was diluted fivefold with nuclease-free water, aliquoted, and immediately stored at − 80 °C until subsequent qPCR analysis. Quantitative real-time reverse transcription PCR (qRT-PCR) was performed via the ChamQ SYBR qPCR Master Mix (Nanjing Vazyme Biotech Co., Ltd.) on a QuantStudio 5 real-time PCR system (Thermo Fisher Scientific) for assessing the relative mRNA expression levels of Fibroblast activation protein (FAP), Cathepsin H (CTSH), MAF Basic Leucine Zipper Transcription Factor F (MAFF), matrix metalloproteinase 1 (MMP1), matrix metalloproteinase 9 (MMP9), and Complement component 6 (C6). The quantification of gene expression was performed through the 2^-ΔΔCt approach, with normalization carried out using the reference gene glyceraldehyde-3-phosphate dehydrogenase (GAPDH). All experiments were conducted independently in triplicate to ensure the reliability of the results.

(The RNA sequence fragments are presented in Table 2.)

**Table 2:** Forward and reverse primer sequences for qRT-PCR

Gene	Forward primer sequence	Reverse primer sequence
FAP	CAAAGGCTGGAGCTAAGAATCC	ACTGCAAACAT ACTCGTTCATCA
CTSH	CCTGTGAAAAATCAGGGTGCC	ATGCATAGGTCCCGGCTCTT
MAFF	TGCCCAGGTCCCATTTCTC	GGCCCACGAAGGGAATGT
MMP1	AAAATTACACGCCAGATTTGCC	GGTGTGACATTACTCCAGAGTTG
MMP9	GGCACCACCACAACATCACCTA	CGGGCAAAGGCGTCGTCAAT
C6	ACCCTCTTCATTCTGCATACC	AGAGCCTCCAGGCCCTTTA
GAPDH	AAGTATGACAACAGCCTCAAG	TCCACGATACCAAAGTTGTC

### Western blot (WB)

Total cellular protein was extracted using radioimmunoprecipitation assay (RIPA) lysis buffer containing phosphatase inhibitors (Beyotime, Shanghai, China). Protein concentrations were determined with the BCA Protein Assay Kit (Thermo Fisher Scientific). Sodium dodecyl sulfate–polyacrylamide gel electrophoresis (SDS-PAGE) gels were prepared, and equal amounts of protein samples (20 µg per lane) were loaded and separated in a Tanon VE-180 vertical electrophoresis chamber under constant voltage (170 V) for 45 min. Proteins were then transferred onto polyvinylidene fluoride (PVDF) membranes using the wet transfer method in a Tanon VE-586 transfer apparatus at a constant current of 600 mA for 30 min. Membranes were blocked in TBST containing 5% skimmed milk at room temperature for 1 h and incubated overnight at 4 °C with the following primary antibodies: anti-FAP (PA5-51057, Thermo Fisher Scientific, 1:500), anti-CTSH (MA5-52536, Thermo Fisher Scientific, 1:1000), anti-MMP1 (MA5-15872, Thermo Fisher Scientific, 1:500), anti-MMP9 (MA5-32705, Thermo Fisher Scientific, 1:5000), anti-C6 (PA5-60527, Thermo Fisher Scientific, 1:250), and anti-MAFF (PA5-85462, Thermo Fisher Scientific, 1:500). The next day, membranes were washed three times with TBST and incubated at room temperature for 1 h with the appropriate secondary antibodies: goat anti-rabbit IgG (H + L), HRP-conjugated (Thermo Fisher Scientific, 1:10,000) or goat anti-mouse IgG (H + L), HRP-conjugated (Thermo Fisher Scientific, 1:5,000). After further washing, signals were visualized using enhanced chemiluminescence (ECL) substrates (Thermo Fisher Scientific) and imaged on a ChemiDoc XRS + system (Bio-Rad). All normal control (NC) and OA samples were analyzed on the same membrane and tested in triplicate to ensure technical consistency.

### Statistical analysis

The data processing and analysis were enabled by RStudio 4.4.2. A two-tailed *p* < 0.05 denoted statistical significance.

## Results

### Identification of DEGs in OA

**Fig. 1:**
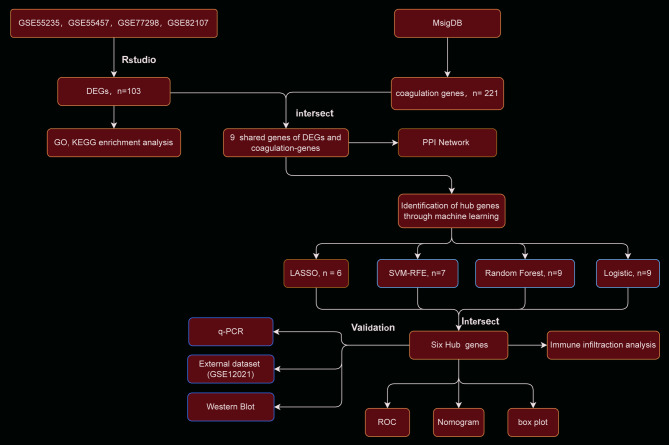
Flow Chart

**Fig. 2:**
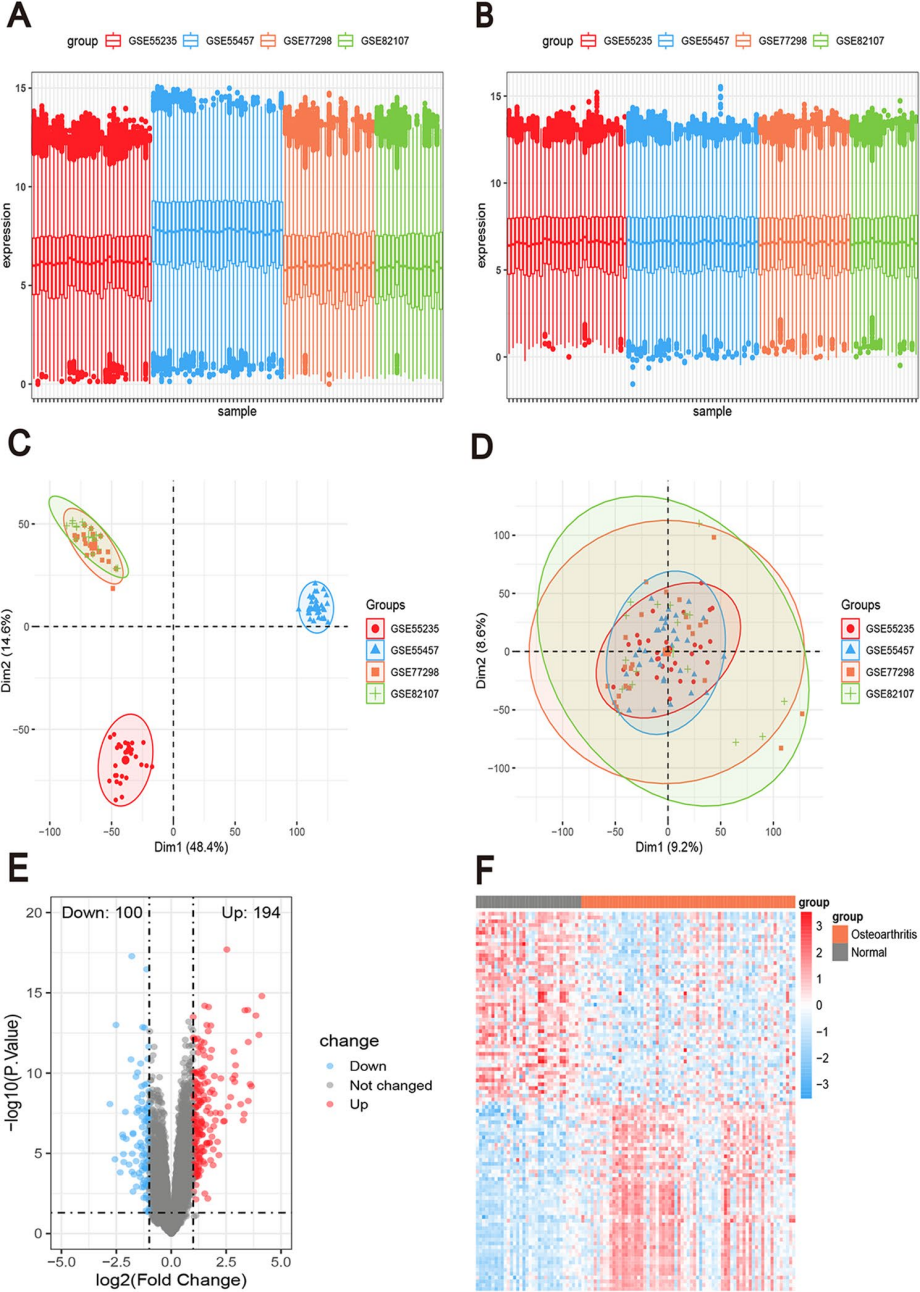
Gene Expression Omnibus(GEO) Data De-Batching. **A** Statistical analysis of gene expression levels in the dataset before batch effect removal. **B** Statistical analysis of gene expression levels in the integrated dataset after batch effect removal. **C** Principal component analysis (PCA) of the dataset before batch effect removal. **D** PCA of the integrated dataset after batch effect removal. **E** Volcano plot of differentially expressed genes (DEGs) associated with OA (|log₂FC| ≥ 1, adjusted *P* < 0.05). **F** Heatmap of DEG expression levels in OA (orange color) and normal control (gray color) samples

The bioinformatics analysis was conducted based on Fig. 1. The GEO dataset was processed to eliminate batch effects, resulting in a consolidated dataset comprising 69 OA and 34 NC samples. As shown in Fig. 2A, substantial heterogeneity was observed in the gene expression profiles across different datasets (GSE55235, GSE55457, GSE77298, GSE82107), manifested as discrepancies in expression levels, indicating a significant batch effect impacting data comparability. Following batch effect correction using the ComBat method, Fig. 2B demonstrates that the distribution of expression profiles across datasets became more uniform, with markedly reduced differences in expression levels. PCA further confirmed the effectiveness of the correction: in Fig. 2C, raw data from different batches exhibited distinct cluster separation patterns in two-dimensional space, reflecting systematic inter-batch variation, whereas in Fig. 2D, the corrected data showed reduced separation and a more compact distribution of data points, indicating successful batch effect removal. DEA revealed 294 DEGs, with 194 showing upregulation and 100 exhibiting downregulation (Fig. 2E). Figure 2F presents a heatmap illustrating the expression patterns of differentially expressed genes between the OA and normal control groups.

### Functional enrichment analysis of DEGs

**Fig. 3:**
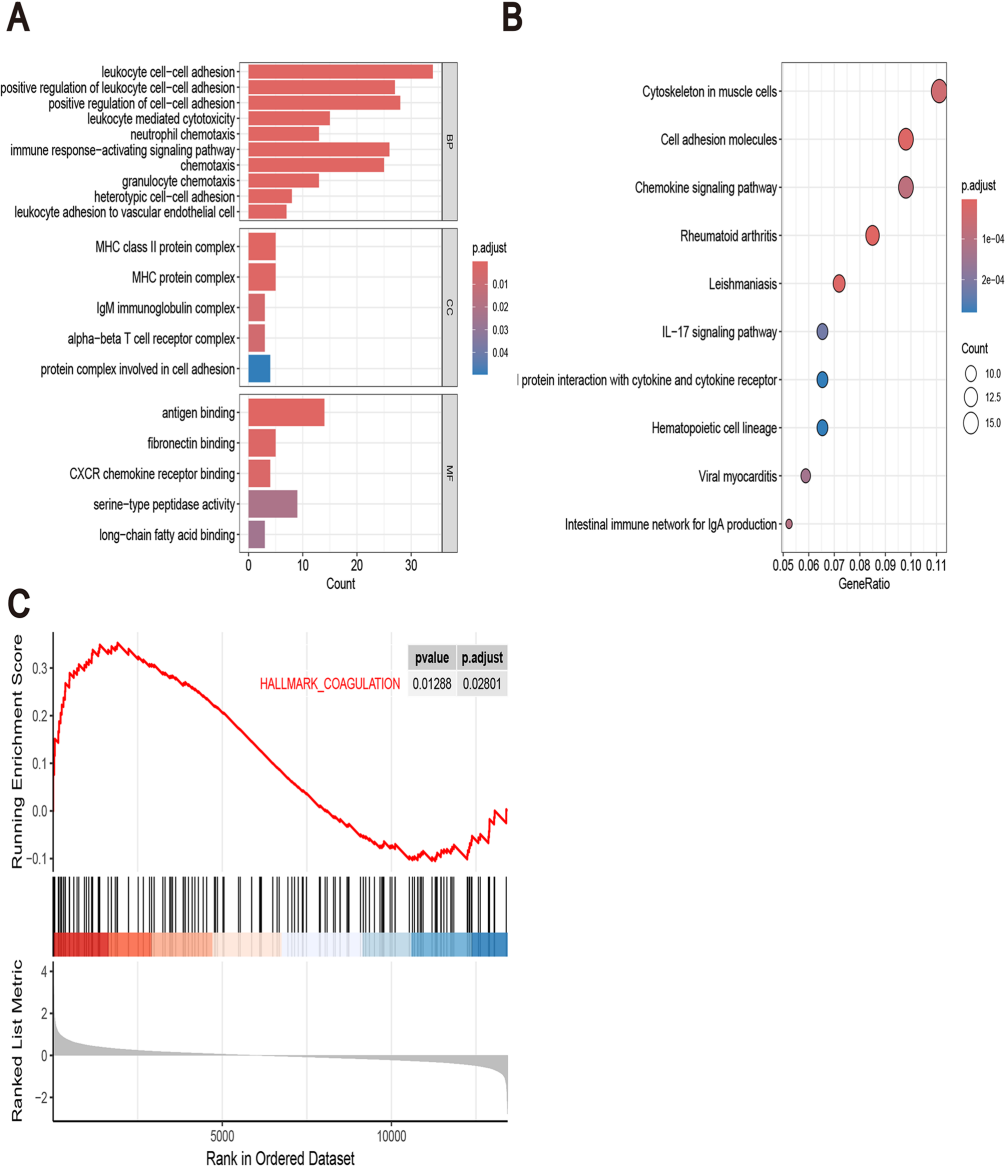
Gene Set Enrichment Analysis (GSEA), Gene Ontology (GO), and Kyoto Encyclopedia of Genes and Genomes (KEGG) Functional Enrichment Analysis of DEGs. **A** Bar plot of GO functional enrichment analysis for DEGs (FDR-adjusted *p* < 0.05) (**B**) Bubble plot of KEGG pathway enrichment analysis for DEGs (FDR-adjusted *p* < 0.05) (**C**) Gene Set Enrichment Analysis of DEGs in the Hallmark Coagulation Gene Set (HALLMARK_COAGULATION from Molecular Signatures Database (MsigDB)) (nominal *p* < 0.05 and FDR q-value < 0.05)

Subsequent GO and KEGG enrichment analyses were conducted using R. Functional annotation of the DEGs revealed significant enrichment in biological processes (BPs) related to cellular immunity, cell adhesion, antigen binding, and fibronectin binding. Notably enriched pathways included leukocyte intercellular adhesion, leukocyte-mediated cytotoxicity, activation of immune responses, and chemotaxis. The Cellular Component (CC) involved included the Major Histocompatibility Complex (MHC) class II protein and IgM immunoglobulin complexes. Molecular functions (MF) were predominantly antigen binding and fibronectin binding(Figure 3A). KEGG pathway analysis identified the most significantly enriched pathways: RA, cell adhesion molecules, cytoskeleton organization, and chemokine signaling pathways (Fig. 3B). The enrichment of cell adhesion processes and fibronectin binding in the results suggests potential involvement in coagulation processes. This inference is supported by the following lines of evidence. Makogonenko et al. demonstrated that fibronectin binds directly and with high affinity to the αC domain of fibrinogen, forming complexes during the coagulation cascade that contribute to the stabilization of thrombus architecture [34]. Furthermore, Midwood et al., using a three-dimensional fibrin–fibronectin model that mimics the provisional wound-healing matrix, showed that fibroblasts engage fibronectin via α5β1 integrins and the transmembrane proteoglycan syndecan-4, leading to rapid formation of focal adhesions and activation of focal adhesion kinase (FAK) and Rho GTPases. This process initiates cytoskeletal remodeling and adhesion signaling. Such a mechanism suggests that, during coagulation, platelets or endothelial cells may similarly exploit fibronectin-enriched matrices to rapidly establish adhesion platforms, thereby amplifying local coagulation signaling [35]. Based on these findings, it is hypothesized that OA is not only associated with inflammatory factors but may also be linked to CRGs. Therefore, an intersection analysis was performed between DEGs and CRGs. GSEA revealed that the DEGs were significantly enriched in the CRG set (HALLMARK_COAGULATION) within the Hallmark canonical pathways (*p* < 0.05), further indicating a strong association between the DEGs and the coagulation process (Fig. 3C).

### Correlation analysis of CRGs

**Fig. 4:**
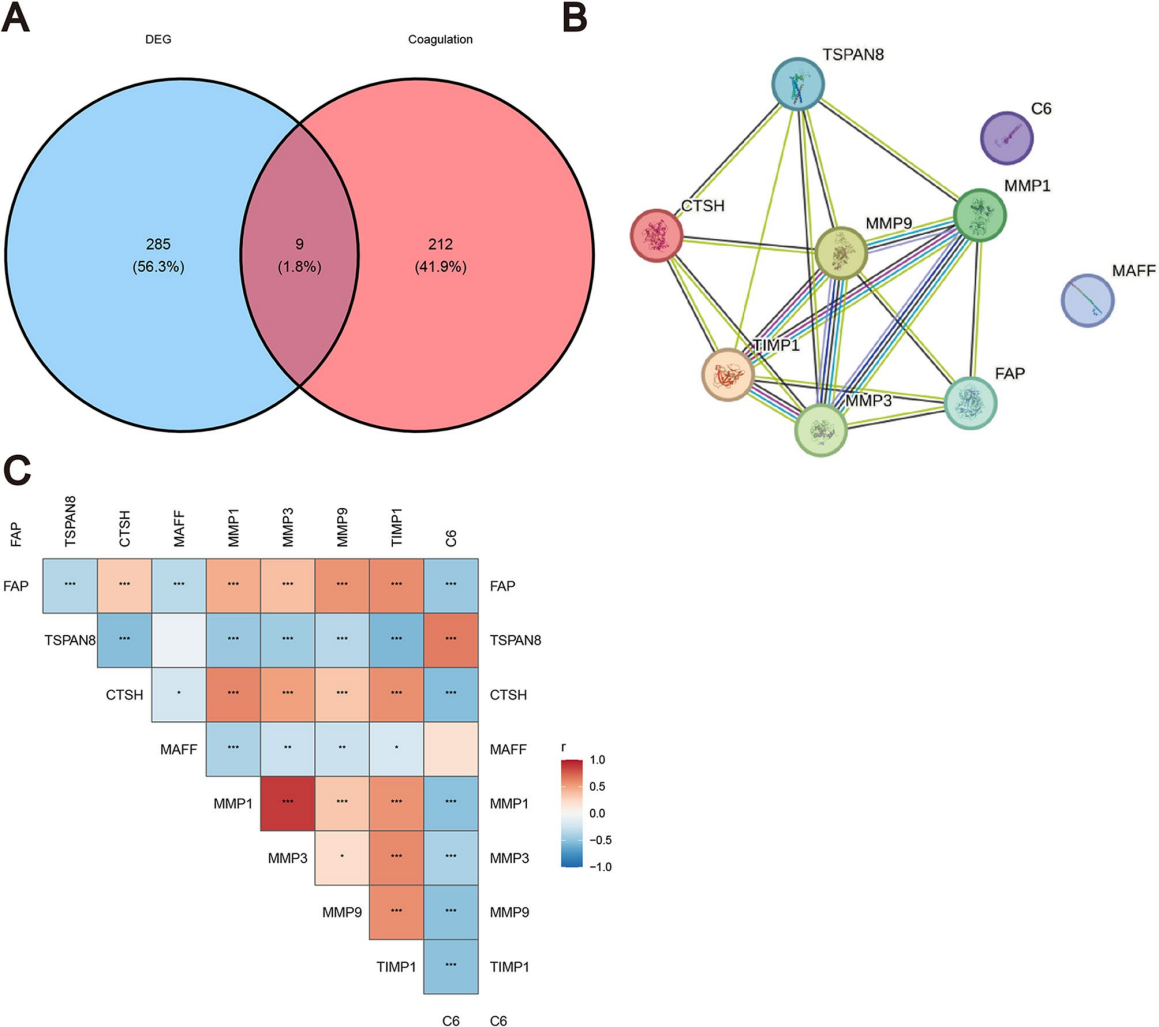
Construction of the Protein-Protein Interaction **(**PPI) Network and Correlation Heatmap of Coagulation-Related Genes (CRGs). **A** Intersection of DEGs and CRGs. **B** PPI network of the intersecting DEGs and CRGs. **C** Correlation heatmap analysis of the intersecting DEGs and CRGs

An intersection analysis identified nine overlapping genes between the 285 DEGs and the 212 CRGs (Fig. 4A). A protein-protein interaction (PPI) network was subsequently constructed for these nine overlapping genes (Fig. 4B). Correlation heatmap visualization demonstrated that the expression levels of these CRGs exhibited significant correlations across different samples (Fig. 4C).

The results demonstrated expression correlations among the nine genes FAP, Tetraspanin-8 (TSPAN8), CTSH, MAFF, MMP1, MMP3, MMP9, Tissue inhibitors of metalloproteinase-1(TIMP1), and C6. The heatmap visually represents Pearson correlation coefficients (r) for gene pairs, ranging from − 1 to 1. Red denotes positive correlations, blue indicates negative correlations, and color intensity reflects the strength of the association. Figure 4C demonstrates that members of the MMP family (MMP1, MMP3, and MMP9) exhibit positive correlations with each other. In contrast, MAFF, TSPAN8, and C6 display predominantly negative correlations with other genes, with the exception that TSPAN8 and C6 are positively correlated with each other. Furthermore, FAP shows negative correlations with both TSPAN8 and C6, while exhibiting positive correlations with all other genes.

### Identification and validation of hub coagulation-related osteoarthritis genes (CROGs)

**Fig. 5:**
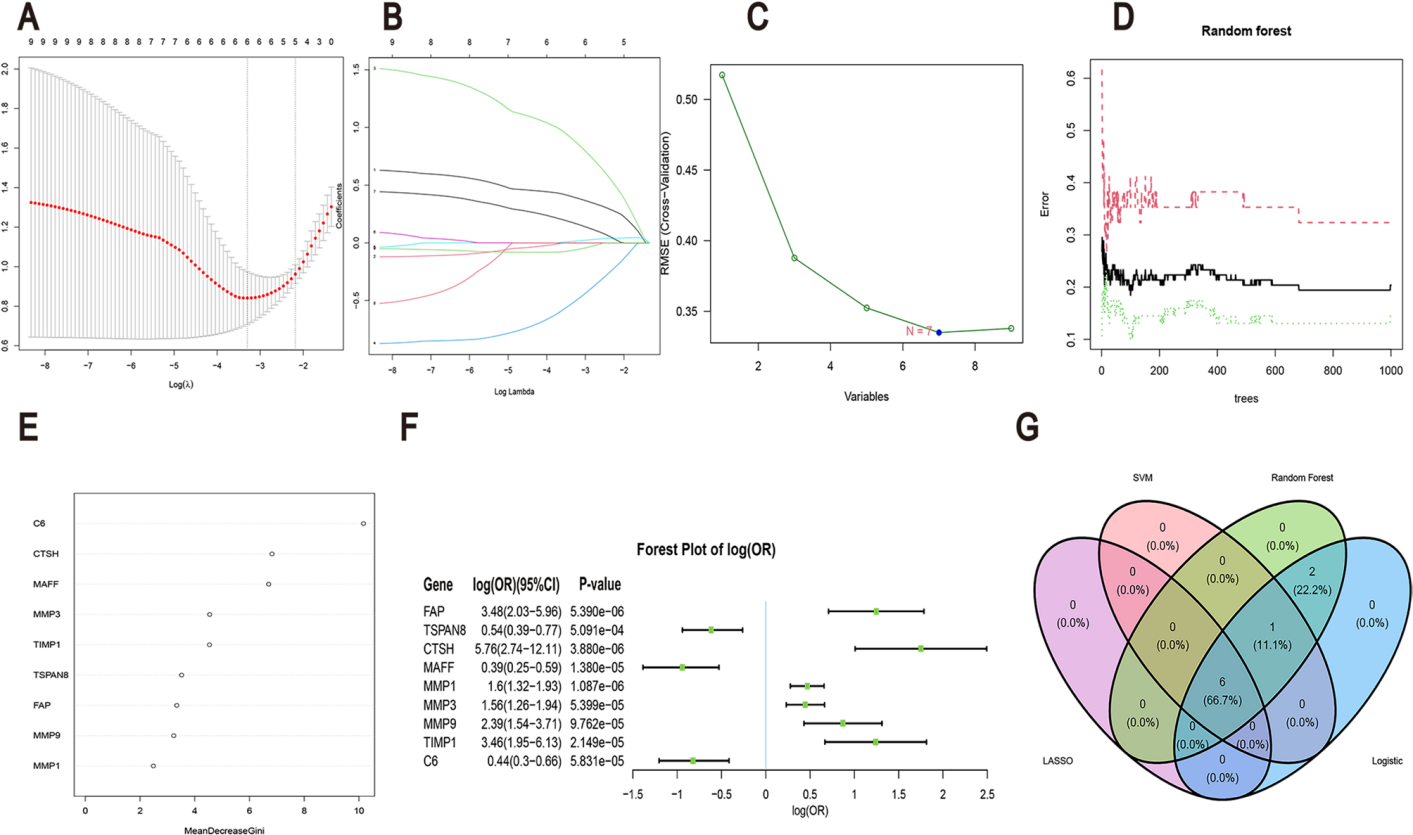
Identification and Validation of Hub CROGs Using four Machine learning (ML) algorithms. **A**, **B** Feature selection of 9 genes using the Least Absolute Shrinkage and Selection Operator (LASSO) algorithm. **C**, **D**, **E** Results of LASSO regression analysis. **F** Forest plot illustrating the expression of 9 genes in OA patients. **G** Venn diagram depicting the intersection of gene selection results from LASSO, Support Vector Machine-Recursive Feature Elimination (SVM-RFE), Random Forest (RF), and logistic regression (LR)

To increase the diagnostic accuracy of CROGs in OA, our study employed four ML algorithms: LASSO (Figs. 5A, B), Support Vector Machine-Recursive Feature Elimination (SVM-RFE) (Figs. 5C, D), RF (Figs. 5E, F), and LR (Fig. 5F) for gene selection. LASSO regression identified *n* = 6 as the optimal number of genes for OA diagnosis, while SVM-RFE indicated that the minimum model error was achieved at *N* = 7. By integrating the results of all four algorithms, six key CROGs were identified: FAP, CTSH, MAFF, MMP1, MMP9, and C6(Fig. 5G).

### Construction of a risk prediction model for hub CROGs

**Fig. 6:**
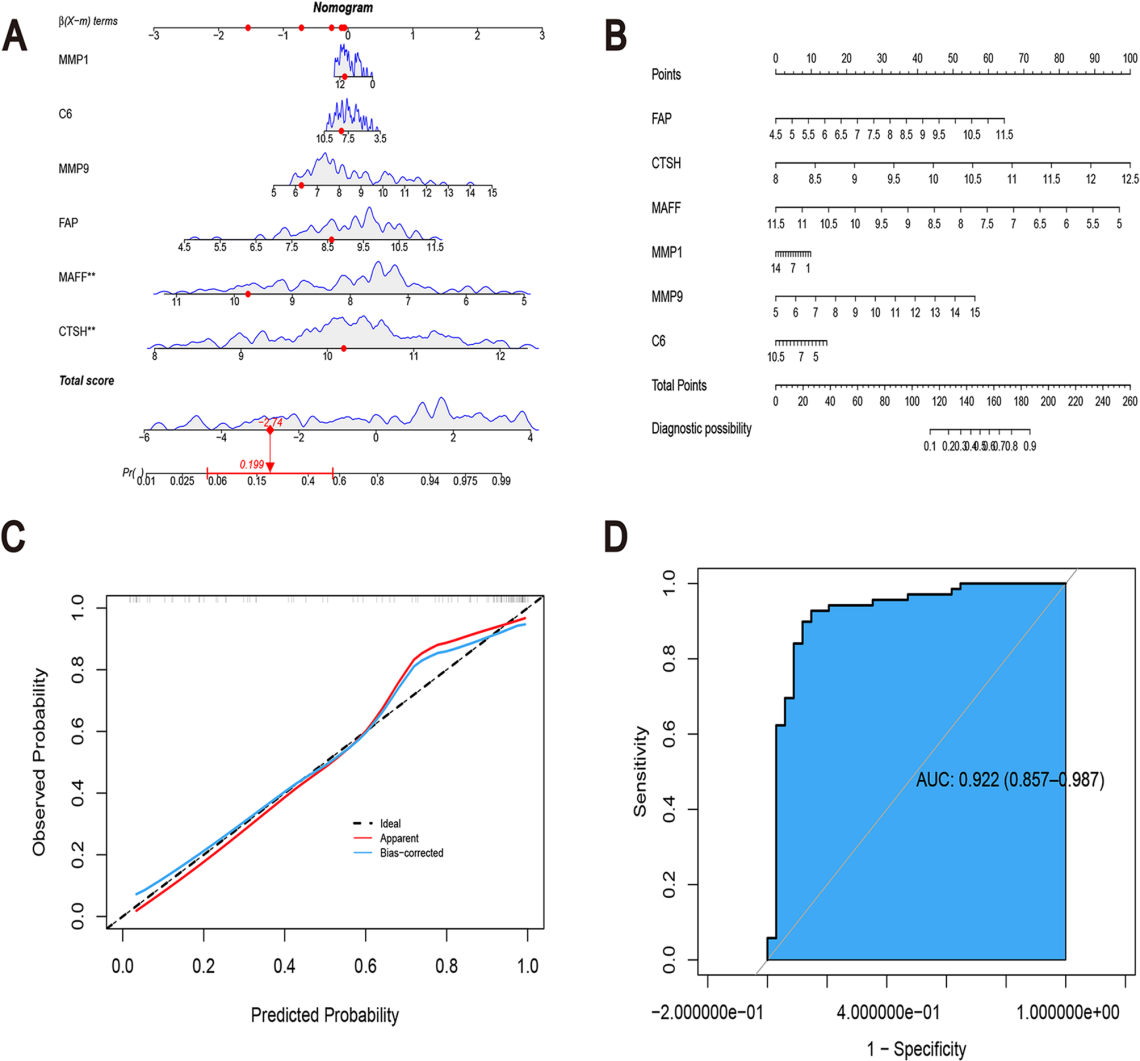
Nomogram plots. **A** Nomogram for predicting OA based on six signature genes. **B** Risk score nomogram for OA diagnosis. **C** Calibration curve, where the dashed line represents an ideal model, the red line indicates the diagnostic model, and the blue line reflects bias-corrected results. **D** Receiver Operating Characteristic (ROC) curve assessing model performance

A nomogram-based OA diagnosis model was developed utilizing the expression profiles of Hub CROGs to enhance clinical applicability. The nomogram for patient diagnosis (Fig. 6A), along with the risk prediction score nomogram (Fig. 6B), ROC curve (Fig. 6D), and calibration curve assessing the concordance between predicted and actual probabilities (Fig. 6C), demonstrated the model’s high performance for OA prediction. The nomogram accurately estimated OA risk corresponding to the expression scores of every gene (Fig. 6A), achieving an AUC value of 0.922 (Fig. 6D), indicating excellent diagnostic efficacy.

### ROC curve analysis of individual genes

**Fig. 7:**
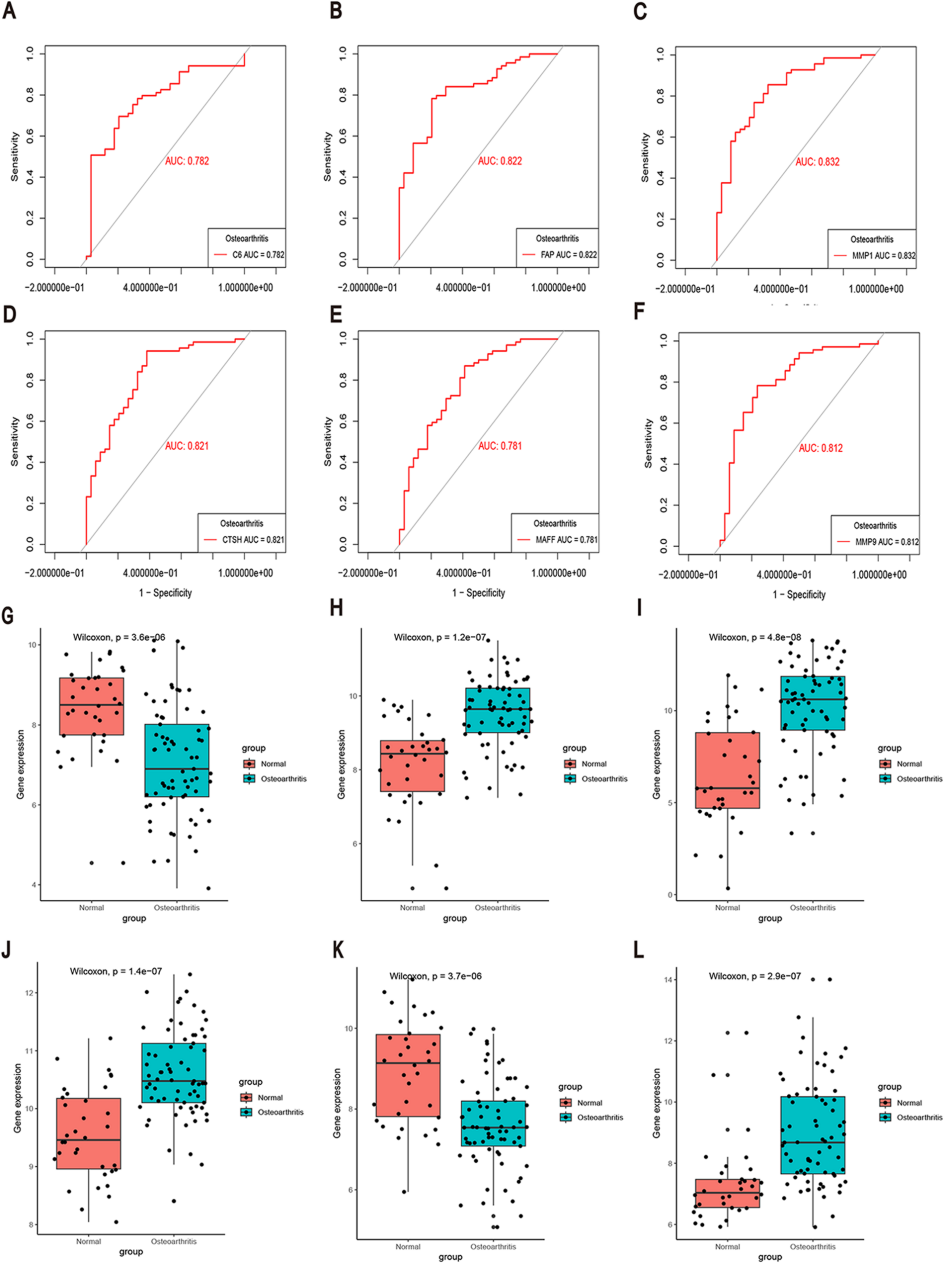
ROC Curve Analysis and Gene Expression of Hub CROGs. **A-F** ROC curves illustrating the diagnostic performance of six genes in OA. **G-L** Box plots depicting the expression of the six genes in OA diagnosis

ROC curve analysis demonstrated that the six hub CROGs and the nomogram exhibited significant diagnostic value for OA in the training cohort. The diagnostic performance of the six genes was as follows (Figs. 7A-F): C6 (AUC = 0.782), FAP (AUC = 0.822), MMP1 (AUC = 0.832), CTSH (AUC = 0.821), MAFF (AUC = 0.781), and MMP9 (AUC = 0.812). The values for all six hub CROGs identified through four ML algorithms exceeded 0.5, indicating their potential as reliable biomarkers for OA diagnosis. Furthermore, box plots depicting the expression levels of these six genes (C6, FAP, MMP1, CTSH, MAFF, and MMP9) were also generated (Figs. 7G-L).

### Validation of key hub genes using the external dataset GSE12021

**Fig. 8:**
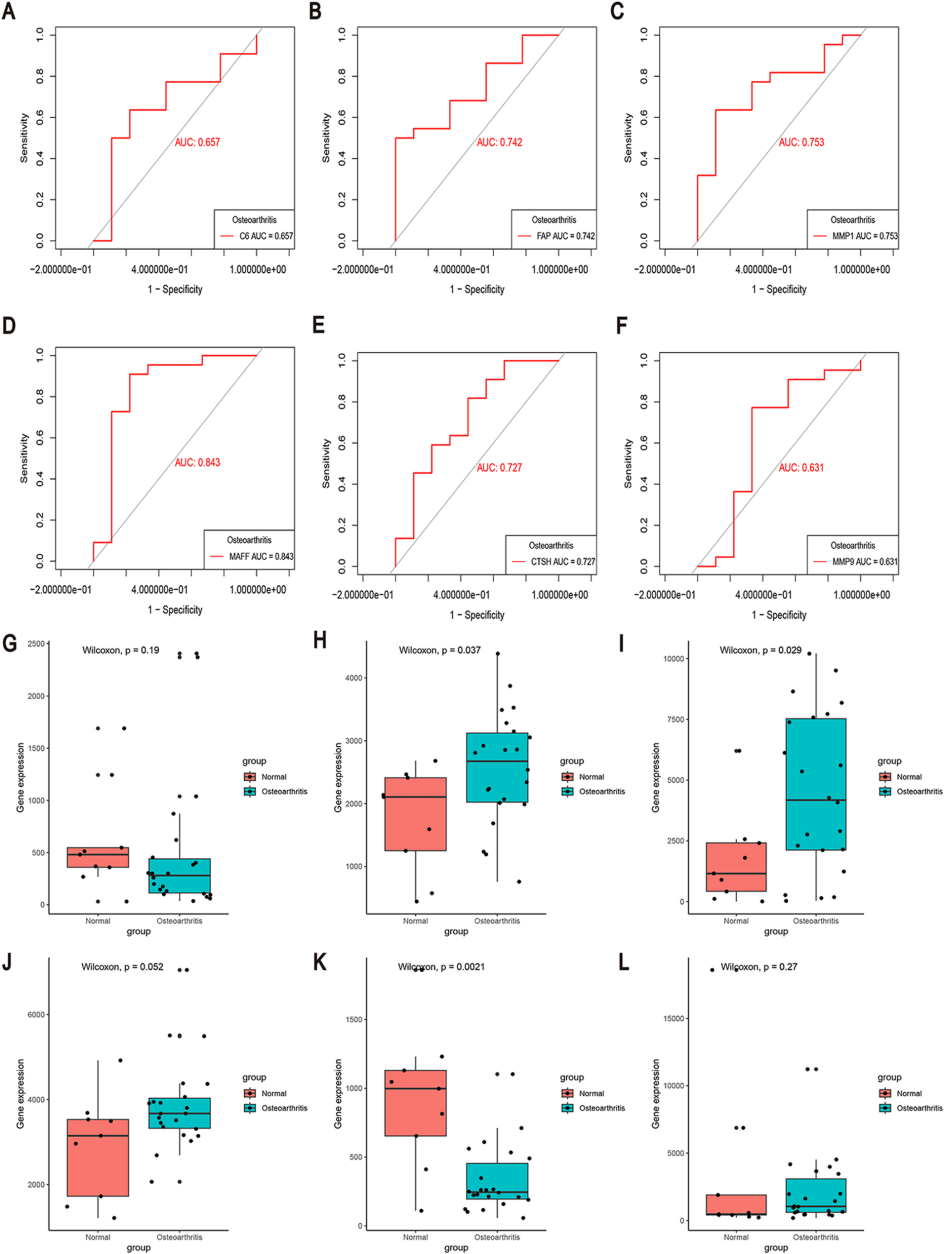
ROC Curve Analysis and Gene Expression of Validation of Key Hub Genes using External Dataset GSE12021. **A-F** ROC curves of six genes in OA diagnosis using the external dataset GSE12021. **G-L** Box plots depicting the expression levels of six genes in OA diagnosis using the external dataset GSE12021

To assess the predictive significance of the six key DEGs, ROC curve analysis was performed using the GSE12021 dataset. All hub genes exhibited AUC values exceeding 0.6 (Figs. 8A–F), with the following AUC values: C6 (0.657), FAP (0.742), MMP1 (0.753), MAFF (0.843), CTSH (0.727), and MMP9 (0.631). Additionally, box plots illustrating the expression levels of these six key DEGs were generated (Figs. 8G–L).

### Immune cell infiltration analysis via CIBERSORTx

**Fig. 9:**
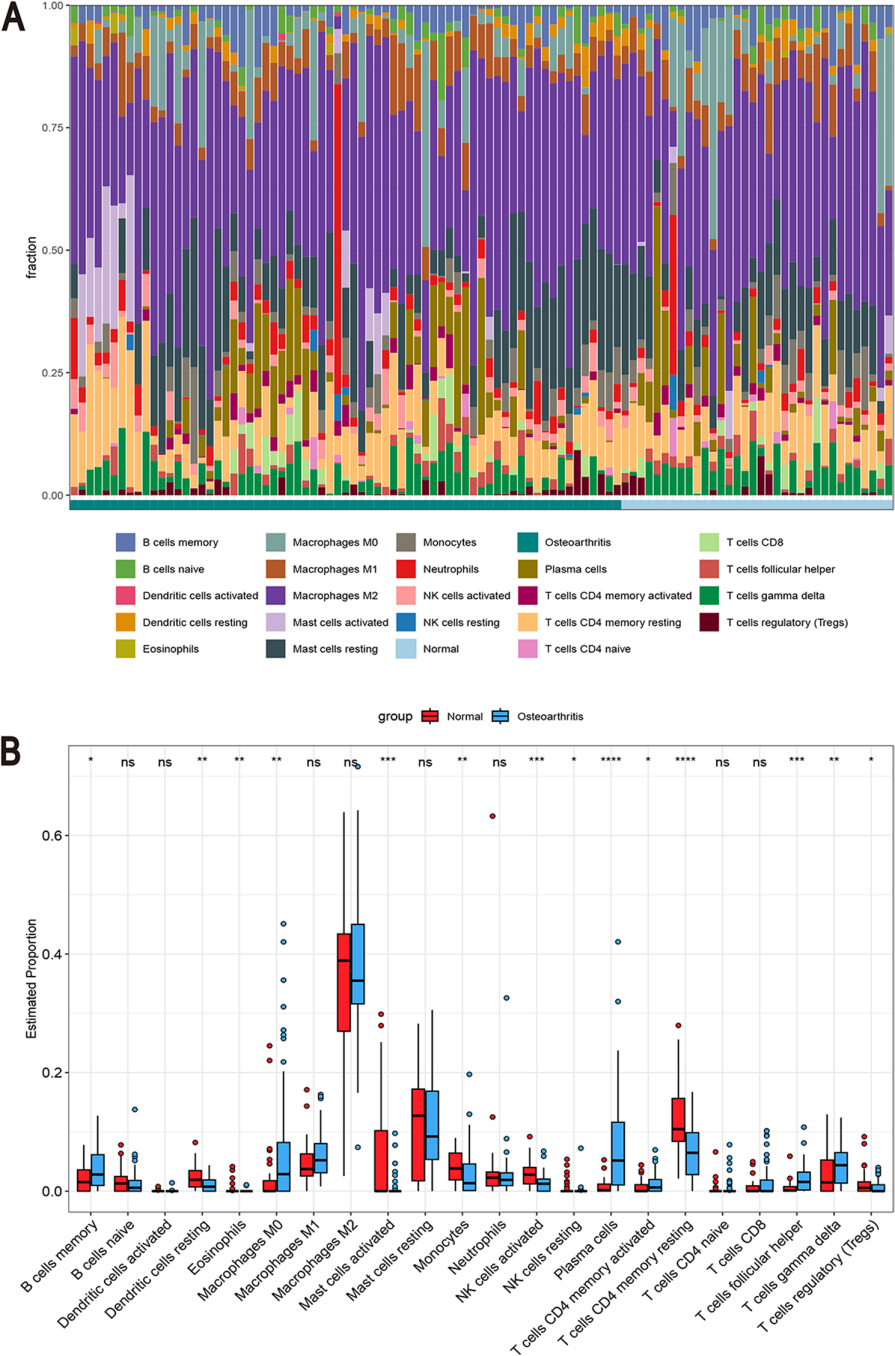
The Results of Immune Cell Infiltration Analysis via CIBERSORTx. **A**) Bar plot illustrating the composition of 22 immune cell types integrated from the GSE5523, GSE77298, GSE82107, and GSE55457 datasets. **B** Box plot showing the differential infiltration levels of the 22 immune cell types

To elucidate the immune cell infiltration characteristics of OA, CIBERSORTx was employed to quantify 22 immune cell types in normal and OA synovial tissues. A stacked bar plot (Fig. 9A) illustrates the distribution of immune cell types across different samples, while a box plot (Fig. 9B) presents the differential abundance of immune cells. In contrast to the normal group, OA synovial tissue exhibited an increased proportion of memory B cells, M0 macrophages, plasma cells, as well as activated memory CD4+, follicular helper, and gamma delta T cells. Conversely, the abundance of monocytes, resting dendritic and memory CD4 + T cells, Tregs, as well as activated mast and NK cells, was decreased.

### qPCR validation of bioinformatics data

**Fig. 10:**
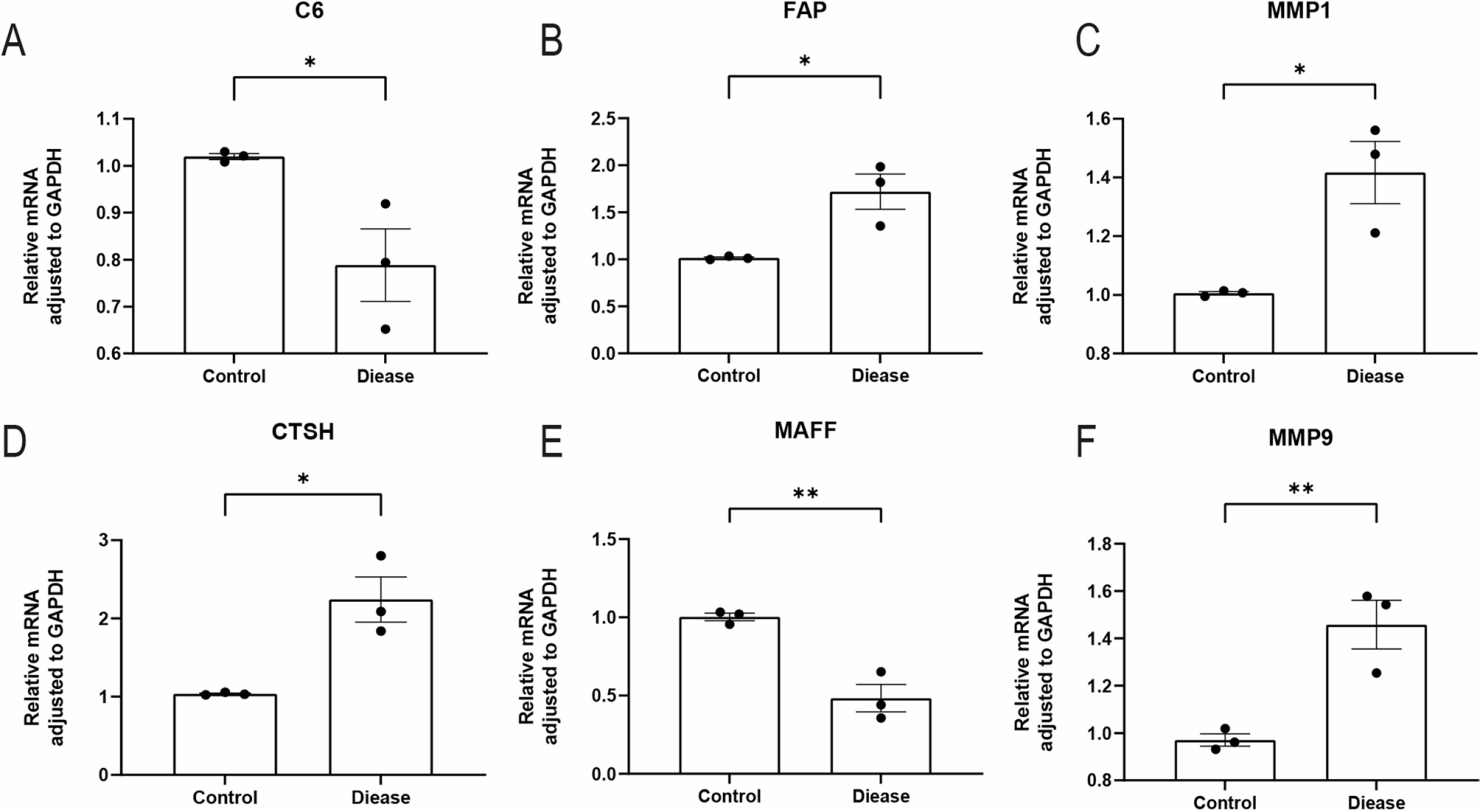
Quantitative real-time reverse transcription PCR (qRT-PCR) validation of differential mRNA expression levels of six diagnostic genes in OA. Panels (A)–(F) show the relative expression levels of C6, FAP, MMP1, CTSH, MAFF, and MMP9, respectively, in normal versus disease groups. *P < 0.05, **P < 0.01

qPCR experiments were carried out to verify the bioinformatics findings. In the IL-1β-treated group, the mRNA expression of FAP, MMP1, CTSH, and MMP9 was notably upregulated, whereas C6 and MAFF expression was markedly downregulated (Fig. 10). Notably, MAFF and MMP9 exhibited the most pronounced differences in expression. The qPCR results aligned with the bioinformatics analysis.

### Validation of bioinformatics findings via WB analysis

**Fig. 11:**
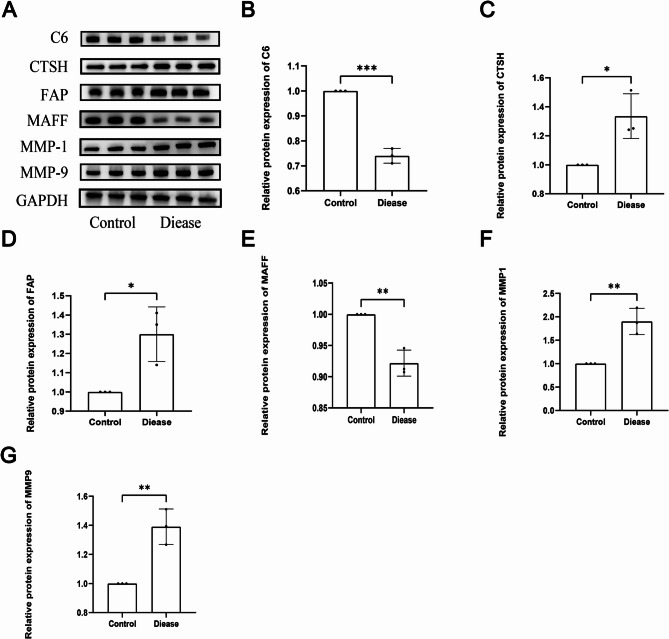
Validation of Differential Protein Expression Levels of Six Diagnostic Genes between Normal and Osteoarthritis Groups by WB Analysis. **A** Western blot (WB) results of the six genes. **B-G **Relative protein expression levels of the six genes in cells from normal and disease groups. Values are presented as mean ± standard deviation. *P < 0.05; **P < 0.01; ***P < 0.001

To further validate the results of the preliminary bioinformatics analysis and qPCR experiments at the protein level, WB assays were carried out. As shown in Fig. 11, the expression levels of the target proteins exhibited marked changes in the IL-1β-stimulated group. Specifically, the protein levels of FAP, MMP1, CTSH, and MMP9 were significantly upregulated, whereas the expression of C6 and MAFF was notably downregulated. These WB results were consistent with the qPCR findings and the outcomes derived from the bioinformatics analysis.

## Discussion

OA is a musculoskeletal disorder with risk factors including age, female sex, obesity, and joint trauma. It is a prevalent and disabling disease that predominantly affects the knee, wrist, and hip joints [36], thereby exacerbating the global burden, impacting approximately 7.6% of the global population [37]. Despite its significant clinical impact, the immunological and coagulation-related molecular mechanisms underlying OA remain insufficiently elucidated. Therefore, identifying reliable biomarkers for the diagnosis, treatment, and prognosis of OA is of great significance. In this study, 103 DEGs were identified between the OA and normal groups (Table 1). Following batch effect correction using the limma package, the combined dataset was intersected with the CRG set from the MsigDB database, yielding nine key CRGs associated with OA (Fig. 4A). A PPI network was then developed via STRING, which revealed interconnections among TSPAN8, CTSH, MMP9, MMP1, FAP, TIMP1, and MMP3 (Fig. 4B). These nine genes were found to overlap with coagulation-related DEGs. GO and KEGG analyses indicated significant enrichment in immune-inflammatory responses, cell adhesion molecules, cytoskeletal organization, chemokine signaling pathways, and coagulation-related pathways (Figs. 3A-B). Four ML algorithms were applied to screen the nine DEGs, and the intersection of these methods identified six key hub genes related to coagulation in OA: FAP, CTSH, MAFF, MMP1, MMP9, and C6 (Figs. 5A-G). The diagnostic utility was evaluated through nomogram models and ROC curves (Figs. 6A-D), with subsequent analysis of immune infiltration (Figs. 9A-B). External validation was performed utilizing the GSE12021 dataset(Figs. 8A-L), and the results were further corroborated via quantitative polymerase chain reaction (qPCR) experiments (Figs. 10A-F).

GO and KEGG analyses of the 103 DEGs demonstrated significant enrichment in BPs such as leukocyte-mediated cytotoxicity, chemotaxis, granulocyte chemotaxis, MHC class II protein complex formation, and CXCR chemokine receptor binding, suggesting that inflammatory responses play a central role in OA pathogenesis. The DEGs were also enriched in pathways related to leukocyte-cell adhesion—a hallmark of inflammation that promotes rapid intercellular contact, monocyte and granulocyte aggregation, adhesion of T lymphocytes to B cells or monocytes, and interactions between leukocytes and vascular endothelial cells [38]. Fibronectin, a glycoprotein essential for cell migration, opsonization, tissue development, and platelet adhesion, was enriched in fibronectin-binding functions [39], indicating a potential role for coagulation in OA progression.

TSPAN8, a member of the tetraspanin superfamily, contains four transmembrane domains and conserved protein motifs. While its role in OA remains unclear, evidence from studies on colorectal cancer suggests that TSPAN8 modulates cell motility by linking epidermal growth factor receptor (EGFR) signaling to integrin/TLR-mediated pathways; EGFR silencing was found to enhance migration in TSPAN8-expressing cells [40]. Similarly, in nasopharyngeal carcinoma, TSPAN8 can facilitate cell migration, invasion, and proliferation through the Akt/MAPK pathway [41]. The foregoing findings suggest that TSPAN8 may participate in the Akt/MAPK pathway, accelerating tissue repair and cell migration.

CTSH encodes a cysteine protease participating in lysosomal protein degradation, activation of additional proteases, and antigen presentation via the MHC class II pathway [42]. In the context of inflammatory cytokines, reduced CTSH expression has been associated with accelerated β-cell destruction in islets via the Rac2 GTPase pathway [43]. Although the role of CTSH in OA remains to be defined, its protective function in other diseases suggests that it may contribute to chondrocyte preservation and antigen presentation during inflammatory responses through the Rac2 pathway.

MMPs, including MMP-1, −2, −3, −9, and − 13, are involved in the degradation of extracellular matrix (ECM) components in articular cartilage [44]. In OA, the levels of leptin in the synovial fluid of OA patients were elevated in contrast to the control group. Moreover, elevated leptin levels in synovial fluid showed a positive correlation with body mass index (BMI), while the expression of both leptin and its receptor (Ob-Rb) was linked to the degree of cartilage degradation. Leptin stimulates the synthesis of MMPs in cartilage via NF-κB, protein kinase C, and MAP kinase pathways [45]. Various stimulatory factors induce the production of different MMPs. For example, TNF stimulates MMP-1, −3, −9, and − 13 production [46]. MMP-3 protein expression level is a significant biomarker for OA detection of OA [47].

FAP of the serine protease family is a significant indicator for detecting tumor-linked fibroblasts in most epithelial cancers [48]. It displays both dipeptidyl peptidase and collagenase activity, enabling the degradation of gelatin and type I collagen [48]. FAP is also capable of degrading type II collagen and is highly expressed in fibroblasts. It plays key roles in tumor microenvironment remodeling, inflammatory responses, and tissue repair, and is thus believed to contribute to OA progression [49]. Moreover, FAP can inhibit osteolectin (Oln/Clec11a/Scgf)-mediated Wnt signaling, thereby suppressing bone formation [50].

Tissue inhibitors of metalloproteinases (TIMPs) are a family of proteins, with four TIMP genes (1–4) present in the human genome. They are extensively distributed in animals [51]and have multiple roles, including acting as inhibitors of MMPs, disintegrin metalloproteinases (ADAMs), and a disintegrin and metalloproteinase with thrombospondin motifs (ADAMTSs) [52].

C6 is the longest protein in the MAC [53], which is the final product of complement activation. C6, a component of MAC, directly causes target cell lysis and death [54]. In a murine model of experimental autoimmune encephalomyelitis (EAE), mice deficient in C5 or C6 exhibited reduced neurological damage and myelin loss, suggesting a critical role for C6 in mediating inflammatory tissue injury [55]. Although the role of C6 in OA remains to be clarified, it may be implicated in cartilage degradation and tissue damage driven by chronic inflammation following soft tissue injury.

MAFF from the MAF oncogene family is crucial in gene regulation, cell differentiation, and tumorigenesis in mammals [56]. In lung cancer, MAFF was shown to promote ferroptosis through the regulation of solute carrier family 7 member 11 (SLC7A11), cyclin-dependent kinase 6 (CDK6), and cyclin-dependent kinase inhibitor 2 C (CDKN2C), thereby inhibiting cell cycle progression from the G1 to S phase and suppressing tumor cell proliferation [57]. In metastatic breast cancer, elevated MAFF expression was associated with activation of the IL-11/signal transducer and activator of transcription 3 (STAT3) pathway, and inhibition of MAFF expression reduced tumor metastasis, suggesting a role in promoting tumor invasion and dissemination [58]. In another study on hepatitis B virus, MAFF suppresses the transcription of the HBV core promoter [59].

However, the specific role and associated pathways of MAFF in the pathogenesis and progression of OA require further experimental validation. Current research has identified the potential roles of these six key hub genes in the prediction of OA. Model evaluation using ROC curves and nomograms demonstrated the model’s good diagnostic performance(Figs. 6A-D). Therefore, these six hub genes could act as promising OA diagnosis biomarkers.

Based on the findings of this study and previously published literature, our study proposes a potential mechanism by which CRGs interact with immune cell activity in OA via the NF-κB/NLRP3 signaling pathways. Inflammatory cytokines, such as members of the interleukin-1 (IL-1) family, can bind to their respective interleukin receptors and activate the NF-κB signaling cascade, which, in turn, induces the production of vascular endothelial growth factor (VEGF) and fibroblast growth factor (FGF) [60]. This pathway also promotes the expression of various pro-inflammatory genes [61], including TNF-α and IL-1β [62], triggering a cascade of downstream responses, such as the upregulation of MMP1 and MMP9. For instance, Areej Al-Roub et al. [63] demonstrated that activation of the NF-κB pathway induces MMP9 expression in monocytes. Moreover, the NF-κB pathway plays a crucial role in regulating the differentiation and effector functions of T cells [64]. In this study, immune infiltration analysis revealed elevated levels of activated memory CD4⁺ T cells, follicular helper T (Tfh) cells, and γδ T cells, suggesting that these alterations may be associated with NF-κB-mediated differentiation of CD4⁺ T cells into various effector subsets. Additionally, the NF-κB pathway can initiate both the transcriptional activation and post-translational modification of the NLRP3 inflammasome [65]. Studies have indicated that the NLRP3 inflammasome acts synergistically with other inflammasomes to promote fibrotic processes [66]. In our study, the upregulation of FAP suggests its potential involvement in fibrosis. Excessive fibrosis may lead to aberrant extracellular matrix deposition, which in turn may activate coagulation pathways. As reported by Giovanni Cenerini et al. [67], pulmonary fibrosis and coagulation can mutually reinforce each other. Furthermore, inflammatory signaling molecules released during coagulation, such as fibrinogen [68], may activate PARs, thereby promoting the release of pro-inflammatory mediators, including cytokines, chemokines, and adhesion molecules, which further exacerbate both inflammatory responses and coagulation cascades [69]. Although no direct evidence has yet established a definitive relationship between CTSH, C6, MAFF, and the processes of coagulation or immune response, literature suggests that CTSH and C6 may be involved in antigen presentation and the initiation of coagulation cascades. Complement component C6 may contribute to immune responses, while MAFF may participate in osteocyte migration and differentiation. Nevertheless, the precise biological functions and signaling mechanisms of these molecules remain incompletely understood and warrant further elucidation through systematic experimental investigations.

Our study fills a critical gap in understanding the interplay between immune responses and coagulation in OA by first investigating the role of CRGs in this context. This novel perspective offers valuable insights into OA pathophysiology and highlights potential new therapeutic targets. Moreover, four distinct ML algorithms were employed to identify biomarkers, with a particular focus on CRGs, an area that remains largely unexplored in prior research.

Nonetheless, certain limitations warrant consideration. The retrospective nature of our study calls for further experimental validation and prospective longitudinal studies to confirm these findings. Additionally, although our study analyzed data from four different datasets, the sample sizes were relatively small, and limited clinical information may constrain a thorough evaluation of individual patient variability and its impact. Moreover, further investigation is necessary to explore the upstream and downstream regulatory pathways linked to the discovered biomarkers and their interrelationships. Finally, this study employed only a single chondrocyte cell line (C28/I2) and IL-1β stimulation, which may not fully recapitulate the complex in vivo osteoarthritic (OA) microenvironment. Future studies should aim to validate these findings in primary chondrocytes or animal models.

In conclusion, our study investigates coagulation-related biomarkers in OA and identifies six potential biomarkers (FAP, CTSH, MAFF, MMP1, MMP9, C6). These genes may represent new potential biomarkers and therapeutic targets for OA. Nevertheless, more experimental studies are necessary to validate their specific roles and mechanisms in OA. To confirm the validity and generalizability of the current findings, there is an urgent need for subsequent studies, particularly longitudinal research designs and in vivo and in vitro experiments, to better understand the expression of these biomarkers across different populations and the mechanisms of the associated pathways. This will enable their prospective application in early OA diagnosis and personalized therapy.

## Data Availability

The datasets GSE55235, GSE7729, and GSE8210 were obtained from the GEO.

## References

[1] Tramś E, Malesa K, Pomianowski S, Kamiński R. Role of platelets in osteoarthritis-updated systematic review and meta-analysis on the role of platelet-rich plasma in osteoarthritis. Cells*. *2022;11(7). 10.3390/cells11071080. 10.3390/cells11071080 PMC8997794 35406644

[2] Cheng Y, Liu J, Su Y, Zhao H, Zhao Y, Wen M, Lu S, Zhang W, Wu J. Clinical impact of coagulation and fibrinolysis markers for predicting postoperative venous thromboembolism in total joint arthroplasty patients. Clin Appl Thromb Hemost. 2019;25:1076029619877458. 31608652 10.1177/1076029619877458 PMC6900621

[3] Tseng S, Reddi AH, Di Cesare PE. Cartilage oligomeric matrix protein (COMP): A biomarker of arthritis. Biomark Insights. 2009;4:33–44. 19652761 10.4137/bmi.s645 PMC2716683

[4] Chen FH, Herndon ME, Patel N, Hecht JT, Tuan RS, Lawler J. Interaction of cartilage oligomeric matrix protein/thrombospondin 5 with Aggrecan. J Biol Chem. 2007;282(34):24591–8. 17588949 10.1074/jbc.M611390200 PMC2905148

[5] Cuéllar VG, Cuéllar JM, Kirsch T, Strauss EJ. Correlation of synovial fluid biomarkers with cartilage pathology and associated outcomes in knee arthroscopy. Arthroscopy. 2016;32(3):475–85. 26524935 10.1016/j.arthro.2015.08.033

[6] Willcockson H, Ozkan H, Chubinskaya S, Loeser RF, Longobardi L. CCL2 induces articular chondrocyte MMP expression through ERK and p38 signaling pathways. Osteoarthr Cartil Open. 2021;3(1):100136. 36475068 10.1016/j.ocarto.2020.100136 PMC9718225

[7] Long AT, Kenne E, Jung R, Fuchs TA, Renné T. Contact system revisited: an interface between inflammation, coagulation, and innate immunity. J Thromb Haemost. 2016;14(3):427–37. 26707513 10.1111/jth.13235

[8] Li X, Sim MMS, Wood JP. Recent insights into the regulation of coagulation and thrombosis. Arterioscler Thromb Vasc Biol. 2020;40(5):e119–25. 32320291 10.1161/ATVBAHA.120.312674 PMC7182067

[9] Zhang QL, Xu Q, Huang R, Sun MZ, Jiang DM, Tao H, Jin H. Thromboelastography maximum amplitude is a valuable biomarker for early atherosclerosis in rheumatoid arthritis patients: a single-center cross-sectional study. Kaohsiung J Med Sci. 2025:e70043. 10.1002/kjm2.70043. 10.1002/kjm2.70043 PMC12412580 40343406

[10] Dijkshoorn B, Hansildaar R, Vedder D, Soutari N, Rudin A, Nordström D, Gudbjornsson B, Lend K, Uhlig T, Haavardsholm EA et al. Impaired coagulation parameters in early RA are restored by effective antirheumatic therapy: a prospective pilot study. RMD Open 2024;10(4). 10.1136/rmdopen-2024-004838. 10.1136/rmdopen-2024-004838 PMC11748942 39740931

[11] Bourguignon A, Tasneem S, Hayward CPM. Update on platelet procoagulant mechanisms in health and in bleeding disorders. Int J Lab Hematol. 2022;44(Suppl 1):89–100. 36074709 10.1111/ijlh.13866

[12] Fahey E, Doyle SL. IL-1 family cytokine regulation of vascular permeability and angiogenesis. Front Immunol. 2019;10:1426. 31293586 10.3389/fimmu.2019.01426 PMC6603210

[13] Burzynski LC, Humphry M, Pyrillou K, Wiggins KA, Chan JNE, Figg N, Kitt LL, Summers C, Tatham KC, Martin PB, et al. The coagulation and immune systems are directly linked through the activation of Interleukin-1α by thrombin. Immunity. 2019;50(4):1033–e10421036. 30926232 10.1016/j.immuni.2019.03.003 PMC6476404

[14] Woetzel D, Huber R, Kupfer P, Pohlers D, Pfaff M, Driesch D, Häupl T, Koczan D, Stiehl P, Guthke R, et al. Identification of rheumatoid arthritis and osteoarthritis patients by transcriptome-based rule set generation. Arthritis Res Ther. 2014;16(2):R84. 24690414 10.1186/ar4526 PMC4060460

[15] Broeren MG, de Vries M, Bennink MB, Arntz OJ, Blom AB, Koenders MI, van Lent PL, van der Kraan PM, van den Berg WB, van de Loo FA. Disease-Regulated gene therapy with Anti-Inflammatory Interleukin-10 under the control of the CXCL10 promoter for the treatment of rheumatoid arthritis. Hum Gene Ther. 2016;27(3):244–54. 26711533 10.1089/hum.2015.127

[16] Broeren MG, de Vries M, Bennink MB, van Lent PL, van der Kraan PM, Koenders MI, Thurlings RM, van de Loo FA. Functional tissue analysis reveals successful cryopreservation of human Osteoarthritic synovium. PLoS ONE. 2016;11(11):e0167076. 27870898 10.1371/journal.pone.0167076 PMC5117761

[17] Ritchie ME, Phipson B, Wu D, Hu Y, Law CW, Shi W. Smyth GK: Limma powers differential expression analyses for RNA-sequencing and microarray studies. Nucleic Acids Res. 2015;43(7):e47. 25605792 10.1093/nar/gkv007 PMC4402510

[18] Law CW, Zeglinski K, Dong X, Alhamdoosh M, Smyth GK, Ritchie ME. A guide to creating design matrices for gene expression experiments. F1000Res. 2020;9:1444. 33604029 10.12688/f1000research.27893.1 PMC7873980

[19] Yu G, Wang LG, Han Y, He QY. ClusterProfiler: an R package for comparing biological themes among gene clusters. Omics. 2012;16(5):284–7. 22455463 10.1089/omi.2011.0118 PMC3339379

[20] Thomas PD, Ebert D, Muruganujan A, Mushayahama T, Albou LP, Mi H. PANTHER: making genome-scale phylogenetics accessible to all. Protein Sci. 2022;31(1):8–22. 34717010 10.1002/pro.4218 PMC8740835

[21] Kanehisa M, Furumichi M, Sato Y, Matsuura Y, Ishiguro-Watanabe M. KEGG: biological systems database as a model of the real world. Nucleic Acids Res. 2025;53(D1):D672–7. 39417505 10.1093/nar/gkae909 PMC11701520

[22] Szklarczyk D, Kirsch R, Koutrouli M, Nastou K, Mehryary F, Hachilif R, Gable AL, Fang T, Doncheva NT, Pyysalo S, et al. The STRING database in 2023: protein-protein association networks and functional enrichment analyses for any sequenced genome of interest. Nucleic Acids Res. 2023;51(D1):D638–46. 36370105 10.1093/nar/gkac1000 PMC9825434

[23] Friedman J, Hastie T, Tibshirani R. Regularization paths for generalized linear models via coordinate descent. J Stat Softw. 2010;33(1):1–22. 20808728 PMC2929880

[24] Lockhart R, Taylor J, Tibshirani RJ, Tibshirani R. A SIGNIFICANCE TEST FOR THE LASSO. Ann Stat. 2014;42(2):413–68. 25574062 10.1214/13-AOS1175 PMC4285373

[25] Zou H, Hastie T. Regularization and variable selection via the elastic net. J Royal Stat Soc Ser B: Stat Methodol. 2005;67(2):301–20.

[26] Oshiro TM, Perez PS, Baranauskas JA. How many trees in a random forest? In: Machine learning and data mining in pattern recognition: 2012// 2012; Berlin, Heidelberg. Springer Berlin Heidelberg: 154–68. 10.1007/978-3-642-31537-4_13.

[27] Breiman L. Random forests. Mach Learn. 2001;45(1):5–32.

[28] Wilcoxon F. Individual comparisons of grouped data by ranking methods. J Econ Entomol. 1946;39:269. 20983181 10.1093/jee/39.2.269

[29] Wilcoxin F. Probability tables for individual comparisons by ranking methods. Biometrics. 1947;3(3):119–22. 18903631

[30] el-Lozy M. The signed-rank (Wilcoxon) test. Lancet. 1969;1(7604):1052. 4181280 10.1016/s0140-6736(69)91849-2

[31] Newman AM, Liu CL, Green MR, Gentles AJ, Feng W, Xu Y, Hoang CD, Diehn M, Alizadeh AA. Robust enumeration of cell subsets from tissue expression profiles. Nat Methods. 2015;12(5):453–7. 25822800 10.1038/nmeth.3337 PMC4739640

[32] Kruskal WH, Wallis WA. Use of ranks in One-Criterion variance analysis. J Am Stat Assoc. 1952;47(260):583–621.

[33] Yang Y-J, Min D-Y, Ren Y-L, Zhang S-Y, Zhang J-Z, Ren L, Wang M-Y, Guan X-F. Transcriptome sequencing reveals the pathogenesis of osteoarthritis in an IL-1β-induced human chondrocyte model. IJP. 2022;18(3):578–87. 10.3923/ijp.2022.578.587.

[34] Makogonenko E, Ingham KC, Medved L. Interaction of the fibronectin COOH-terminal Fib-2 regions with fibrin: further characterization and localization of the Fib-2-binding sites. Biochemistry. 2007;46(18):5418–26. 17425334 10.1021/bi7001373 PMC2531210

[35] Midwood KS, Mao Y, Hsia HC, Valenick LV, Schwarzbauer JE. Modulation of cell-fibronectin matrix interactions during tissue repair. J Investig Dermatol Symp Proc. 2006;11(1):73–8. 17069013 10.1038/sj.jidsymp.5650005

[36] Hunter DJ, Bierma-Zeinstra S. Osteoarthritis. Lancet. 2019;393(10182):1745–59. 10.1016/S0140-6736(19)30417-9 31034380

[37] Courties A, Kouki I, Soliman N, Mathieu S, Sellam J. Osteoarthritis year in review 2024: epidemiology and therapy. Osteoarthritis Cartilage. 2024;32(11):1397–404. 39103081 10.1016/j.joca.2024.07.014

[38] Patarroyo M, Prieto J, Rincon J, Timonen T, Lundberg C, Lindbom L, Asjö B, Gahmberg CG. Leukocyte-cell adhesion: a molecular process fundamental in leukocyte physiology. Immunol Rev. 1990;114:67–108. 1973408 10.1111/j.1600-065x.1990.tb00562.x

[39] Forsyth J, Plow EF, Ginsberg MH. Fibronectin binding to platelets. Methods Enzymol. 1992;215:311–6. 1435331 10.1016/0076-6879(92)15073-l

[40] Zhu Y, Ailane N, Sala-Valdés M, Haghighi-Rad F, Billard M, Nguyen V, Saffroy R, Lemoine A, Rubinstein E, Boucheix C, et al. Multi-factorial modulation of colorectal carcinoma cells motility - partial coordination by the tetraspanin Co-029/tspan8. Oncotarget. 2017;8(16):27454–70. 28418857 10.18632/oncotarget.16247 PMC5432348

[41] Lin X, Bi Z, Hu Q, Li Q, Liu J, Luo ML, Xiang Y, Yao H. TSPAN8 serves as a prognostic marker involving Akt/MAPK pathway in nasopharyngeal carcinoma. Ann Transl Med. 2019;7(18):470. 31700906 10.21037/atm.2019.08.02 PMC6803210

[42] Turk B, Turk D, Turk V. Protease signalling: the cutting edge. Embo J. 2012;31(7):1630–43. 22367392 10.1038/emboj.2012.42 PMC3321211

[43] Fløyel T, Mirza AH, Kaur S, Frørup C, Yarani R, Størling J, Pociot F. The Rac2 GTPase contributes to cathepsin H-mediated protection against cytokine-induced apoptosis in insulin-secreting cells. Mol Cell Endocrinol. 2020;518:110993. 32814070 10.1016/j.mce.2020.110993

[44] Burrage PS, Brinckerhoff CE. Molecular targets in osteoarthritis: metalloproteinases and their inhibitors. Curr Drug Targets. 2007;8(2):293–303. 17305507 10.2174/138945007779940098

[45] Koskinen A, Vuolteenaho K, Nieminen R, Moilanen T, Moilanen E. Leptin enhances MMP-1, MMP-3 and MMP-13 production in human Osteoarthritic cartilage and correlates with MMP-1 and MMP-3 in synovial fluid from OA patients. Clin Exp Rheumatol. 2011;29(1):57–64. 21345293

[46] Murphy G, Nagase H. Reappraising metalloproteinases in rheumatoid arthritis and osteoarthritis: destruction or repair? Nat Clin Pract Rheumatol. 2008;4(3):128–35. 18253109 10.1038/ncprheum0727

[47] Lohmander LS, Brandt KD, Mazzuca SA, Katz BP, Larsson S, Struglics A, Lane KA. Use of the plasma Stromelysin (matrix metalloproteinase 3) concentration to predict joint space narrowing in knee osteoarthritis. Arthritis Rheum. 2005;52(10):3160–7. 16200596 10.1002/art.21345

[48] Huber MA, Kraut N, Park JE, Schubert RD, Rettig WJ, Peter RU, Garin-Chesa P. Fibroblast activation protein: differential expression and Serine protease activity in reactive stromal fibroblasts of melanocytic skin tumors. J Invest Dermatol. 2003;120(2):182–8. 12542520 10.1046/j.1523-1747.2003.12035.x

[49] Kou X, Xu X, Chen C, Sanmillan ML, Cai T, Zhou Y, Giraudo C, Le A, Shi S. The Fas/Fap-1/Cav-1 complex regulates IL-1RA secretion in mesenchymal stem cells to accelerate wound healing. Sci Transl Med. 2018;10(432). 10.1126/scitranslmed.aai8524. 10.1126/scitranslmed.aai8524 PMC6310133 29540618

[50] Wei H, Xu Y, Wang Y, Xu L, Mo C, Li L, Shen B, Sun Y, Cheng P, Yang L, et al. Identification of fibroblast activation protein as an osteogenic suppressor and Anti-osteoporosis drug target. Cell Rep. 2020;33(2):108252. 33053358 10.1016/j.celrep.2020.108252

[51] Brew K, Nagase H. The tissue inhibitors of metalloproteinases (TIMPs): an ancient family with structural and functional diversity. Biochim Biophys Acta. 2010;1803(1):55–71. 20080133 10.1016/j.bbamcr.2010.01.003 PMC2853873

[52] Nagase HM. G: The Cancer Degradome; 2008. https://link.springer.com/book/10.1007/978-0-387-69057-5

[53] Aleshin AE, Schraufstatter IU, Stec B, Bankston LA, Liddington RC, DiScipio RG. Structure of complement C6 suggests a mechanism for initiation and unidirectional, sequential assembly of membrane attack complex (MAC). J Biol Chem. 2012;287(13):10210–22. 22267737 10.1074/jbc.M111.327809 PMC3323040

[54] Qiao F, Atkinson C, Song H, Pannu R, Singh I, Tomlinson S. Complement plays an important role in spinal cord injury and represents a therapeutic target for improving recovery following trauma. Am J Pathol. 2006;169(3):1039–47. 16936276 10.2353/ajpath.2006.060248 PMC1698827

[55] Sewell DL, Nacewicz B, Liu F, Macvilay S, Erdei A, Lambris JD, Sandor M, Fabry Z. Complement C3 and C5 play critical roles in traumatic brain cryoinjury: blocking effects on neutrophil extravasation by C5a receptor antagonist. J Neuroimmunol. 2004;155(1–2):55–63. 15342196 10.1016/j.jneuroim.2004.06.003 PMC4766842

[56] Kannan MB, Solovieva V, Blank V. The small MAF transcription factors MAFF, MAFG and MAFK: current knowledge and perspectives. Biochim Biophys Acta. 2012;1823(10):1841–6. 22721719 10.1016/j.bbamcr.2012.06.012

[57] Liang J, Bi G, Huang Y, Zhao G, Sui Q, Zhang H, Bian Y, Yin J, Wang Q, Chen Z, et al. MAFF confers vulnerability to cisplatin-based and ionizing radiation treatments by modulating ferroptosis and cell cycle progression in lung adenocarcinoma. Drug Resist Updat. 2024;73:101057. 38266355 10.1016/j.drup.2024.101057

[58] Moon EJ, Mello SS, Li CG, Chi JT, Thakkar K, Kirkland JG, Lagory EL, Lee IJ, Diep AN, Miao Y, et al. The HIF target MAFF promotes tumor invasion and metastasis through IL11 and STAT3 signaling. Nat Commun. 2021;12(1):4308. 34262028 10.1038/s41467-021-24631-6 PMC8280233

[59] Ibrahim MK, Abdelhafez TH, Takeuchi JS, Wakae K, Sugiyama M, Tsuge M, Ito M, Watashi K, El Kassas M, Kato T, et al. MafF is an antiviral host factor that suppresses transcription from hepatitis B virus core promoter. J Virol. 2021;95(15):e0076721. 33980595 10.1128/JVI.00767-21 PMC8274605

[60] Wang R, Ma Y, Zhan S, Zhang G, Cao L, Zhang X, Shi T, Chen W. B7-H3 promotes colorectal cancer angiogenesis through activating the NF-κB pathway to induce VEGFA expression. Cell Death Dis. 2020;11(1):55. 31974361 10.1038/s41419-020-2252-3 PMC6978425

[61] Liu T, Zhang L, Joo D, Sun SC. NF-κB signaling in inflammation. Signal Transduct Target Ther. 2017;2:17023–. 29158945 10.1038/sigtrans.2017.23 PMC5661633

[62] Verhelst K, Carpentier I, Beyaert R. Regulation of TNF-induced NF-κB activation by different cytoplasmic ubiquitination events. Cytokine Growth Factor Rev. 2011;22(5–6):277–86. 22119011 10.1016/j.cytogfr.2011.11.002

[63] Al-Roub A, Akhter N, Al-Rashed F, Wilson A, Alzaid F, Al-Mulla F, Sindhu S, Ahmad R. TNFα induces matrix metalloproteinase-9 expression in monocytic cells through ACSL1/JNK/ERK/NF-kB signaling pathways. Sci Rep. 2023;13(1):14351. 37658104 10.1038/s41598-023-41514-6 PMC10474281

[64] Chang JH, Xiao Y, Hu H, Jin J, Yu J, Zhou X, Wu X, Johnson HM, Akira S, Pasparakis M, et al. Ubc13 maintains the suppressive function of regulatory T cells and prevents their conversion into effector-like T cells. Nat Immunol. 2012;13(5):481–90. 22484734 10.1038/ni.2267 PMC3361639

[65] Swanson KV, Deng M, Ting JP. The NLRP3 inflammasome: molecular activation and regulation to therapeutics. Nat Rev Immunol. 2019;19(8):477–89. 31036962 10.1038/s41577-019-0165-0 PMC7807242

[66] Artlett CM. The mechanism and regulation of the NLRP3 inflammasome during fibrosis. Biomolecules. 2022;12(5). 10.3390/biom12050634. 10.3390/biom12050634 PMC9138796 35625564

[67] Cenerini G, Chimera D, Pagnini M, Bazzan E, Conti M, Turato G, Celi A, Neri T. The intricate relationship between pulmonary fibrosis and thrombotic pathology: a narrative review. Cells. 2024;13(24). 10.3390/cells13242099. 10.3390/cells13242099 PMC11674501 39768190

[68] van der Meijden PEJ, Heemskerk JWM. Platelet biology and functions: new concepts and clinical perspectives. Nat Rev Cardiol. 2019;16(3):166–79. 30429532 10.1038/s41569-018-0110-0

[69] Wilhelm G, Mertowska P, Mertowski S, Przysucha A, Strużyna J, Grywalska E, Torres K. The crossroads of the coagulation system and the immune system: interactions and connections. Int J Mol Sci. 2023;24(16). 10.3390/ijms241612563. 10.3390/ijms241612563 PMC10454528 37628744

